# Supplementary material for: Bi‐allelic pathogenic variants in NDUFC2 cause early‐onset Leigh syndrome and stalled biogenesis of complex I
Source: EMBO Mol Med. 2020 Sep 24;12(11):e12619. doi: 10.15252/emmm.202012619 (PMC7645371; doi:10.15252/emmm.202012619)
Supplement: Supplementary file 1 — Expanded View Figures PDF [file EMMM-12-e12619-s001.pdf]

Expanded View Figures

Figure EV1. Neuroimaging of Subjects 1, 2 and 3 reveals signal abnormalities suggestive of mitochondrial disease involvement.

A Axial T2-weighted imaging of Subject 1 at 21 months showed bilateral hyperintense signals in the pons (arrows).
B Coronal T2-weighted imaging of Subject 1 at 21 months showed bilateral hyperintense signals in the thalami and substantia nigra (arrows).
C Axial T2-weighted imaging of Subject 2 at 10 days of age showed bilateral hyperintense signals in the basal ganglia (arrows).
D Sagittal T2 imaging of Subject 2 at 10 days of age showed Dandy-Walker malformation and thinning of the corpus callosum (arrows).
E, F Axial and coronal (respectively) T2-weighted imaging of Subject 3 at 5 months of age showed bilateral hyperintense signals in the thalami (arrows).

© 2020 The Authors

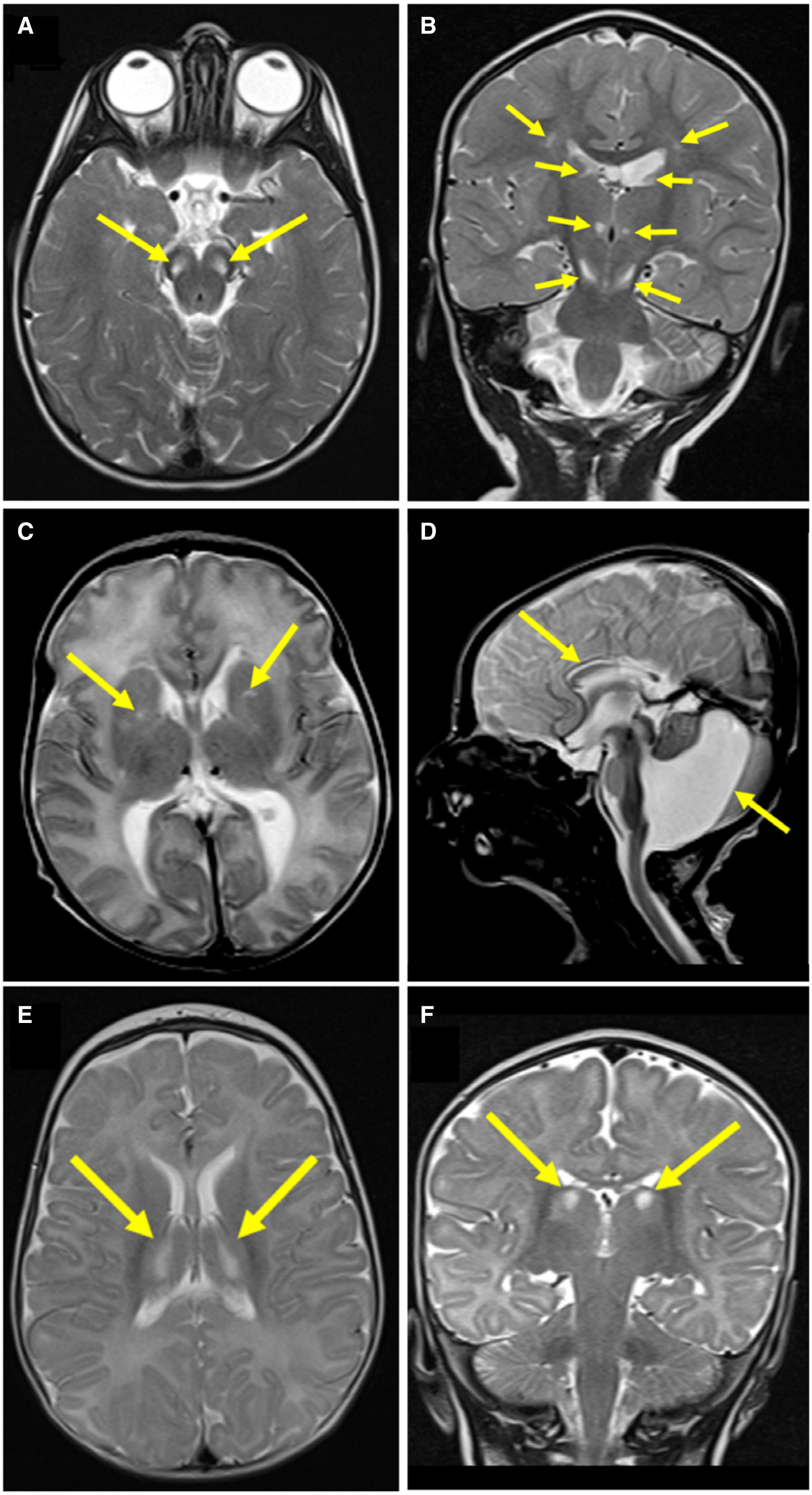

Figure EV1.

© 2020 The Authors

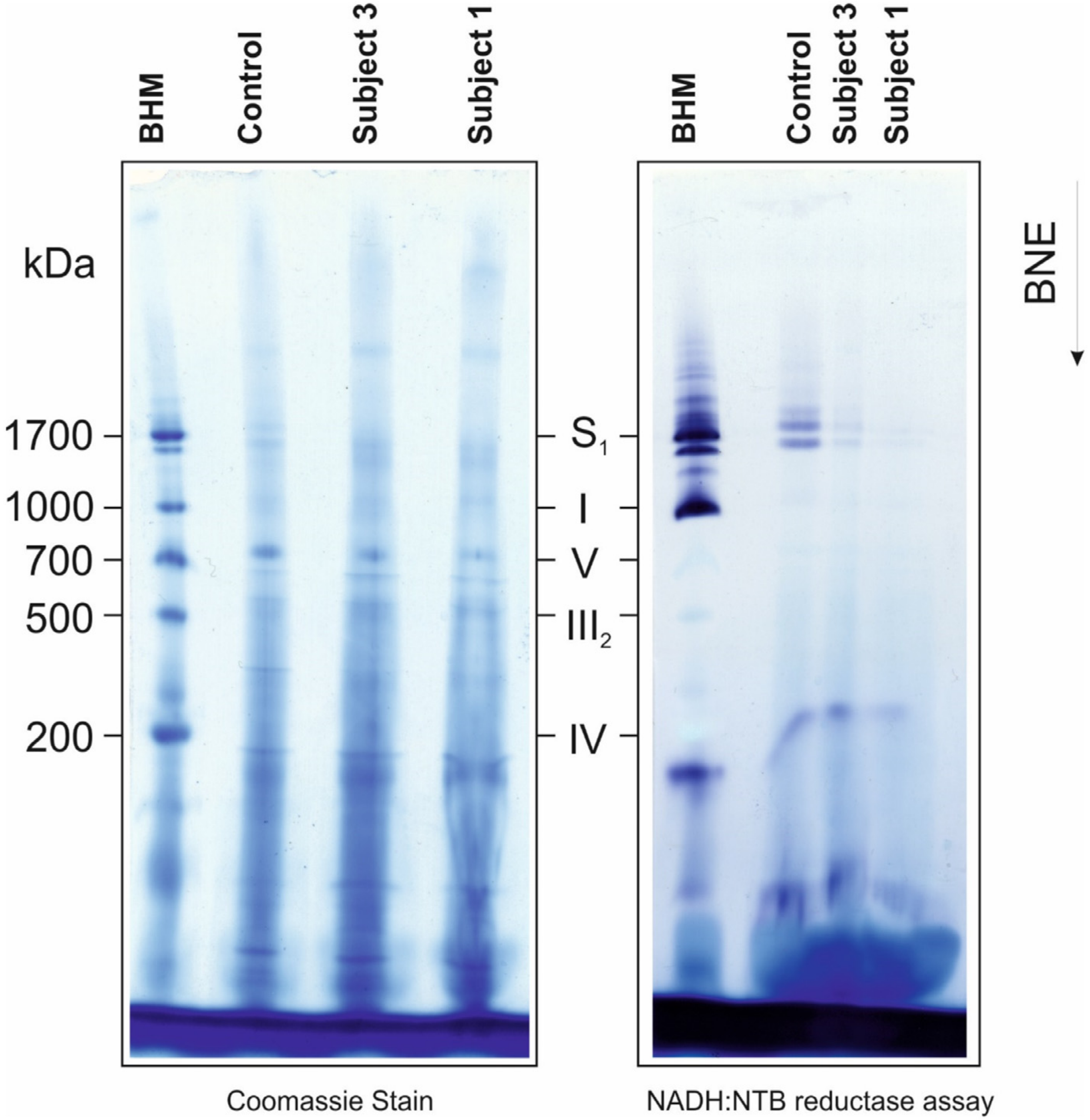

Figure EV2. *NDUFC2* variants cause a severe reduction of complex I-containing supercomplexes.

Coomassie stain (left panel) and an in-gel complex I assay (right panel) in BN-PAGE showed a reduction of complex I-containing supercomplexes in subject mitochondria. Analysis of complex assembly and stability detected a drastic deficiency of complex I-containing supercomplexes.

S_1, supercomplex containing complex I, dimer of complex III and 1 copy of complex IV; V, ATP synthase; I-III-IV, complexes I-III-IV. BHM, bovine heart mitochondria solubilised with digitonin as native mass ladder.

© 2020 The Authors
