# Supplementary material for: Bi‐allelic pathogenic variants in NDUFC2 cause early‐onset Leigh syndrome and stalled biogenesis of complex I
Source: EMBO Mol Med. 2020 Sep 24;12(11):e12619. doi: 10.15252/emmm.202012619 (PMC7645371; doi:10.15252/emmm.202012619)

Figure 3A

NDUFC2 panel

Chemiluminescent signal used in figure

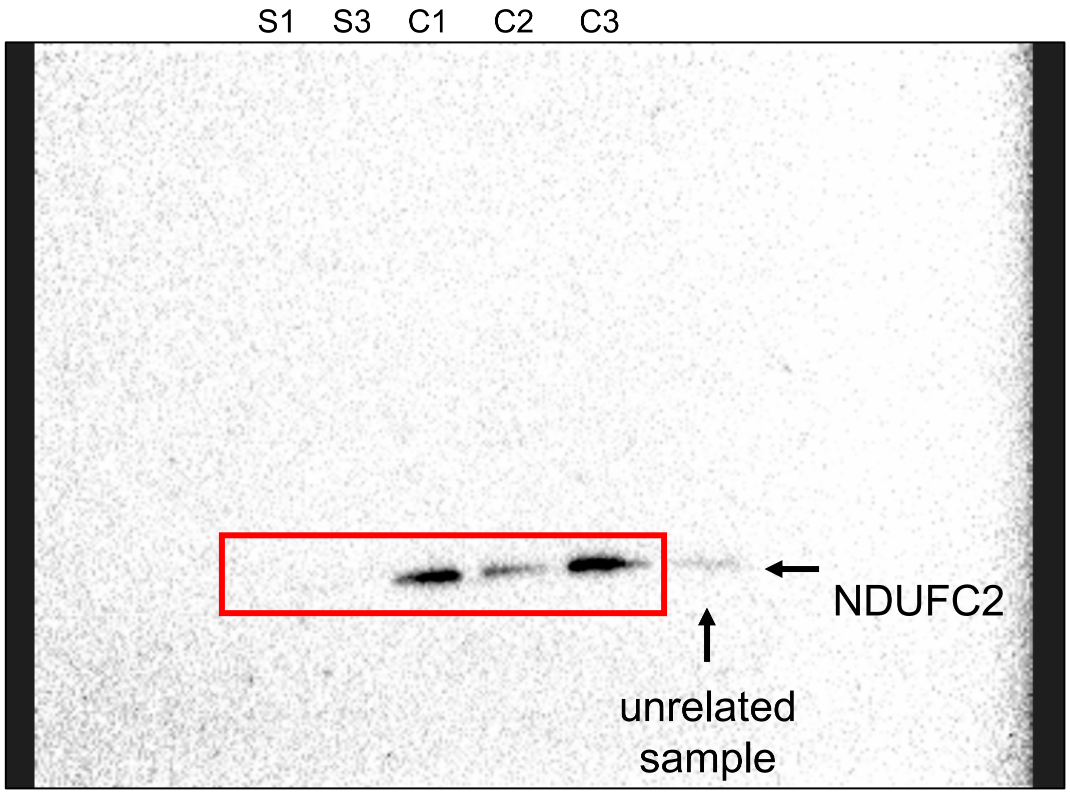

Anti-NDUFC2

Colourimetric image to show MW marker (kDa)

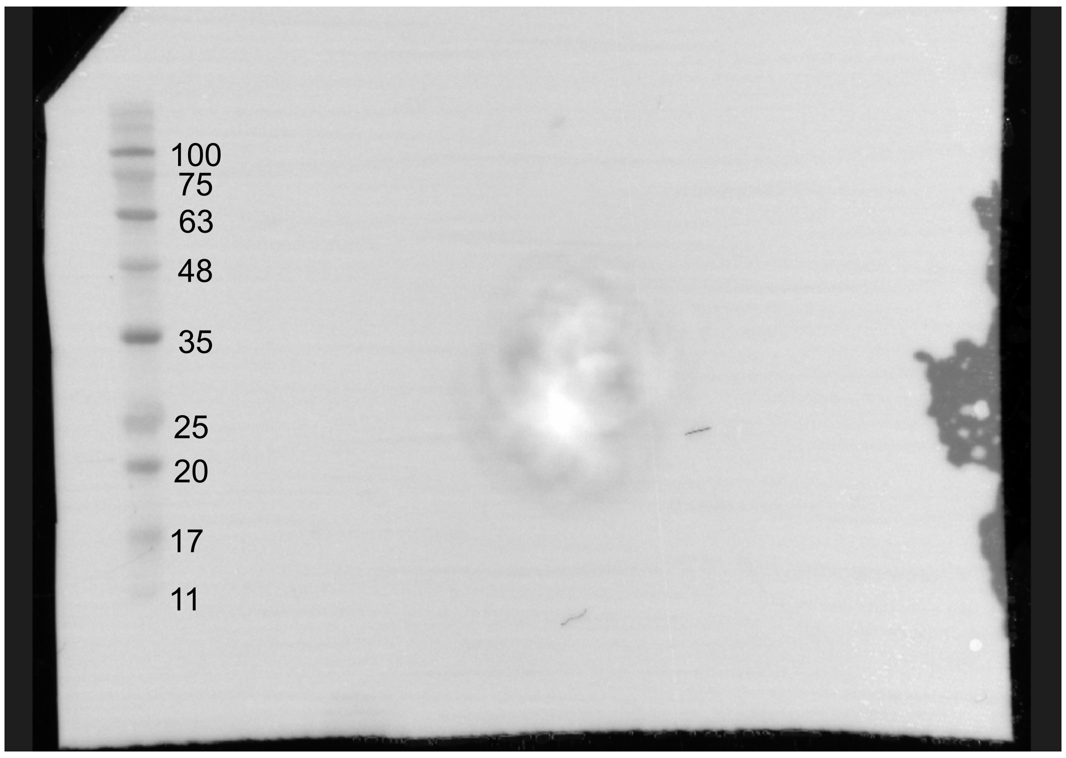

Merged image

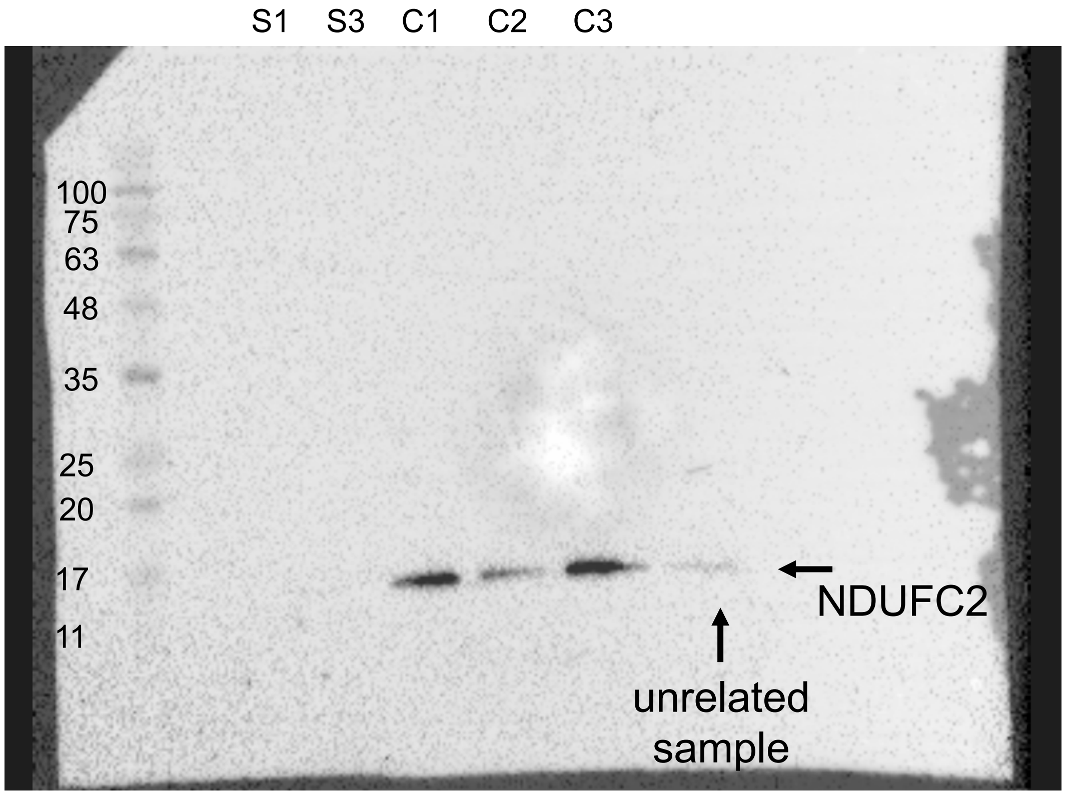

Figure 3A continued

NDUFA9 panel

Chemiluminescent signal used in figure

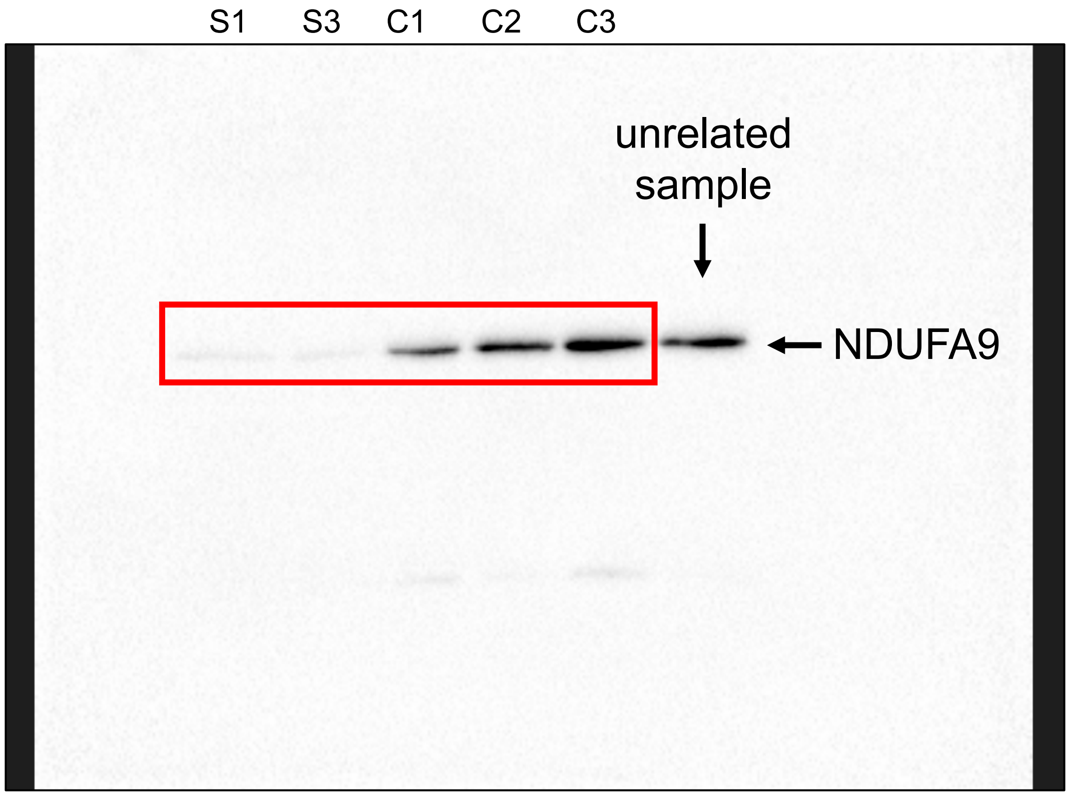

Colourimetric image to show MW marker (kDa)

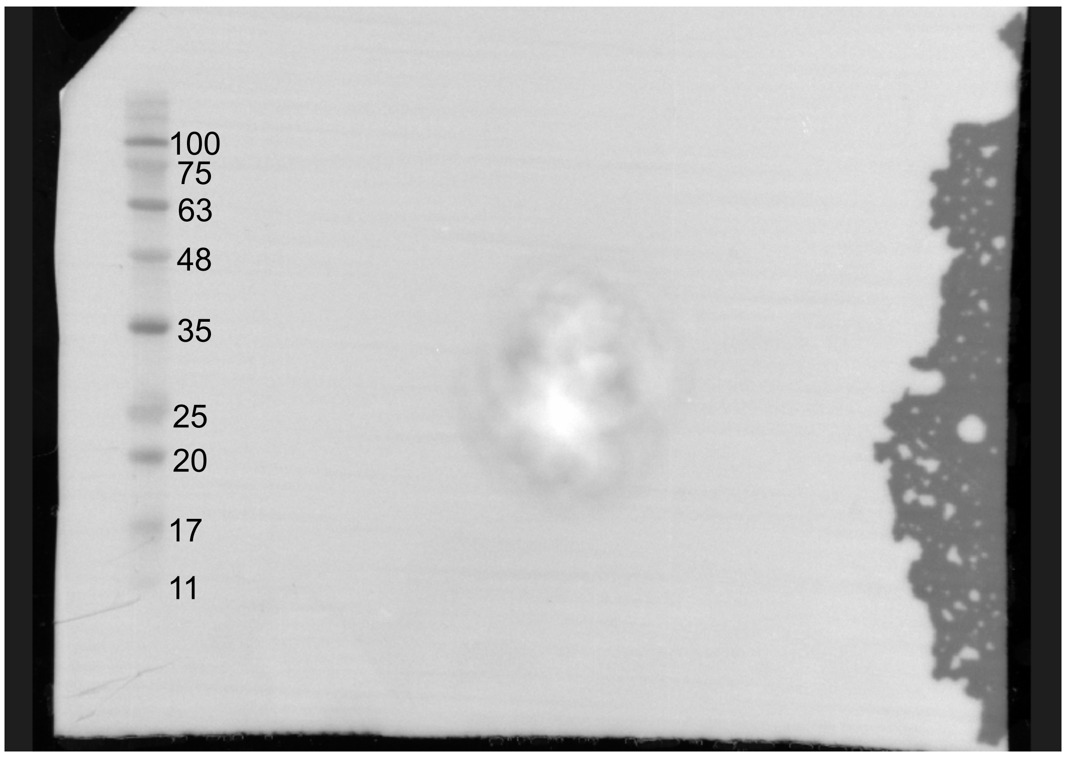

Merged image

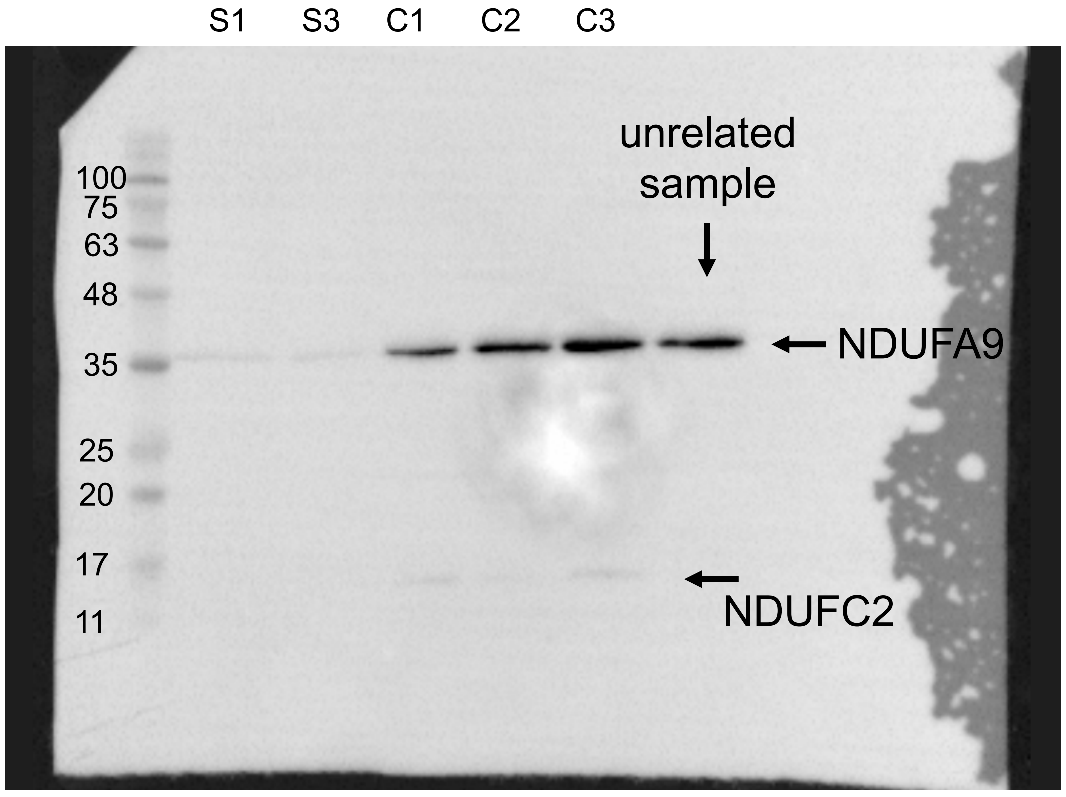

Figure 3A continued

NDUFV1 panel

Chemiluminescent signal used in figure

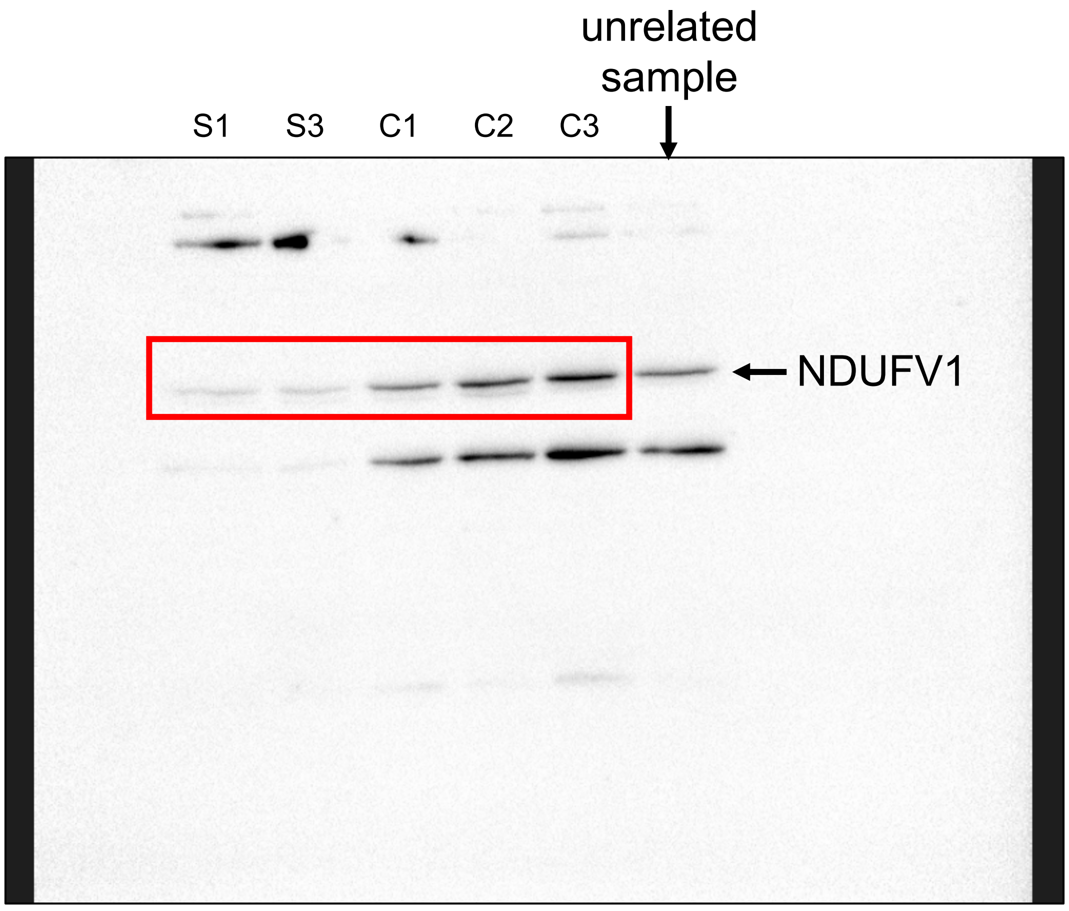

Colourimetric image to show MW marker (kDa)

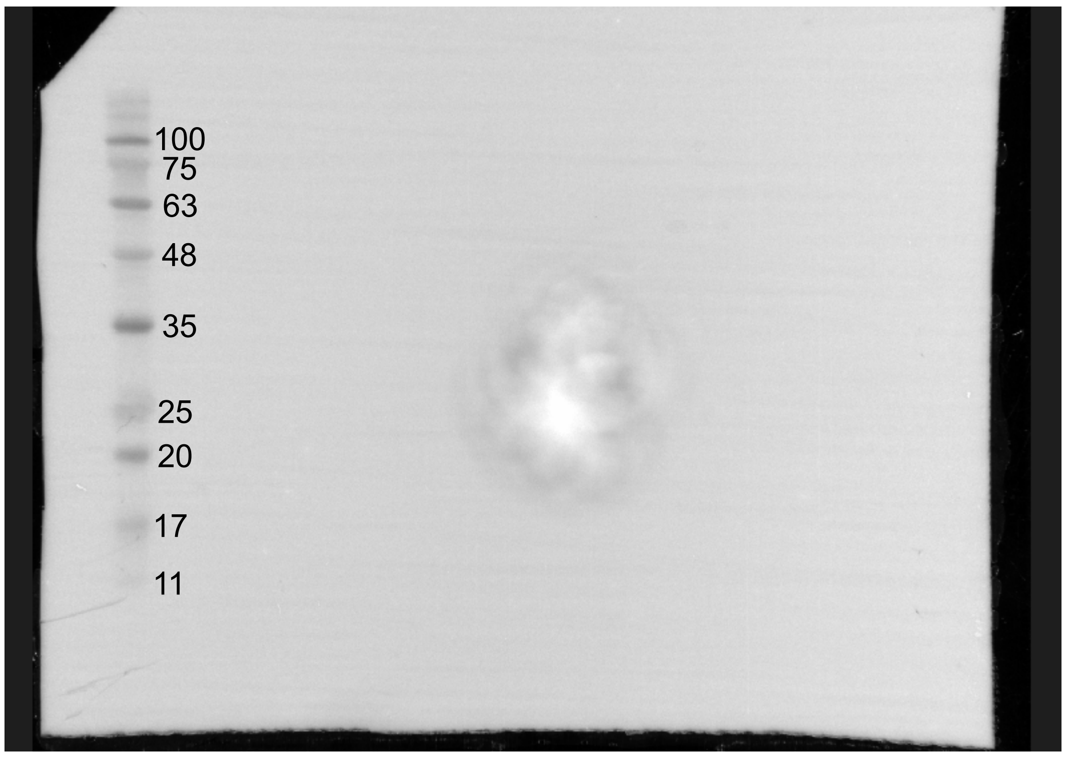

Merged image

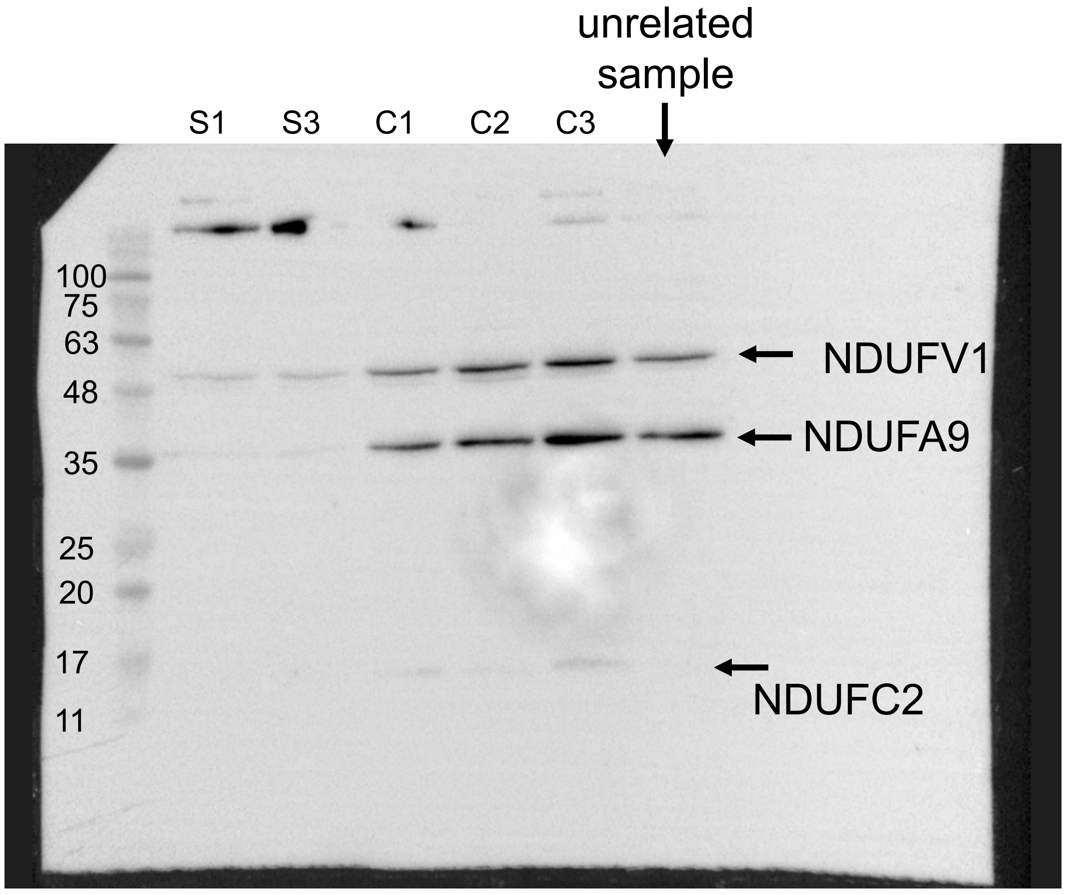

Figure 3A continued

SDHA panel

Chemiluminescent signal used in figure

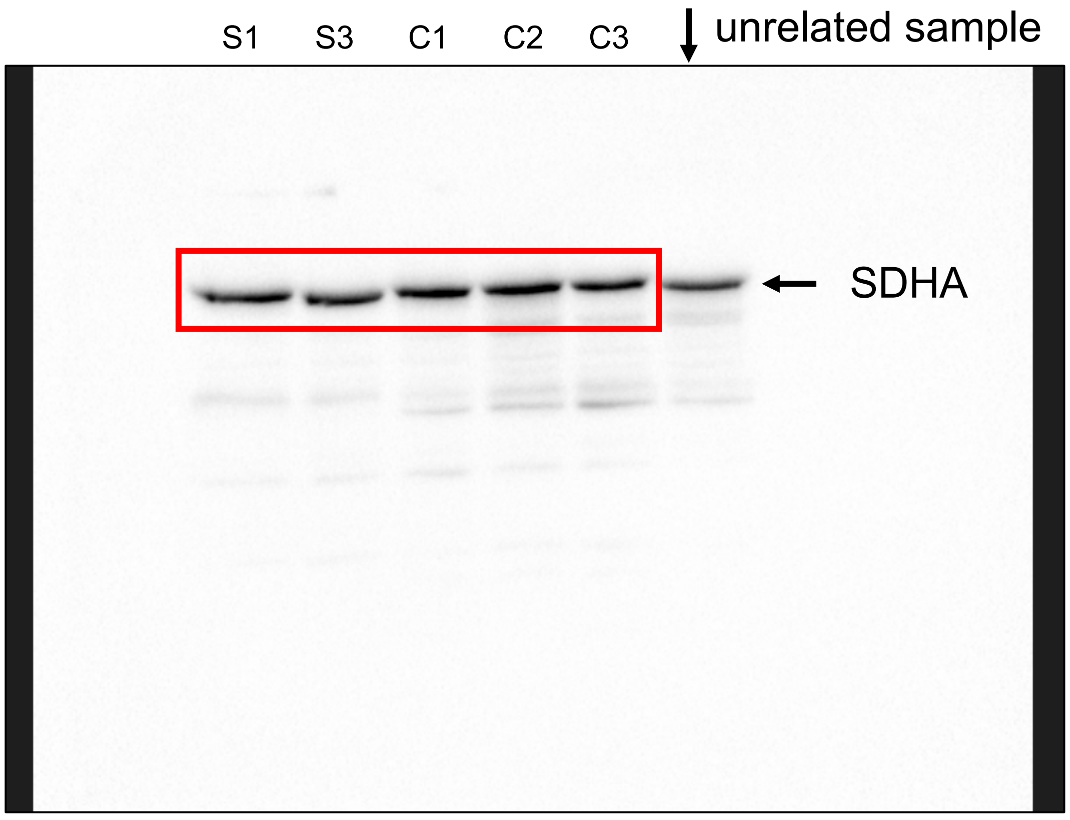

Colourimetric image to show MW marker (kDa)

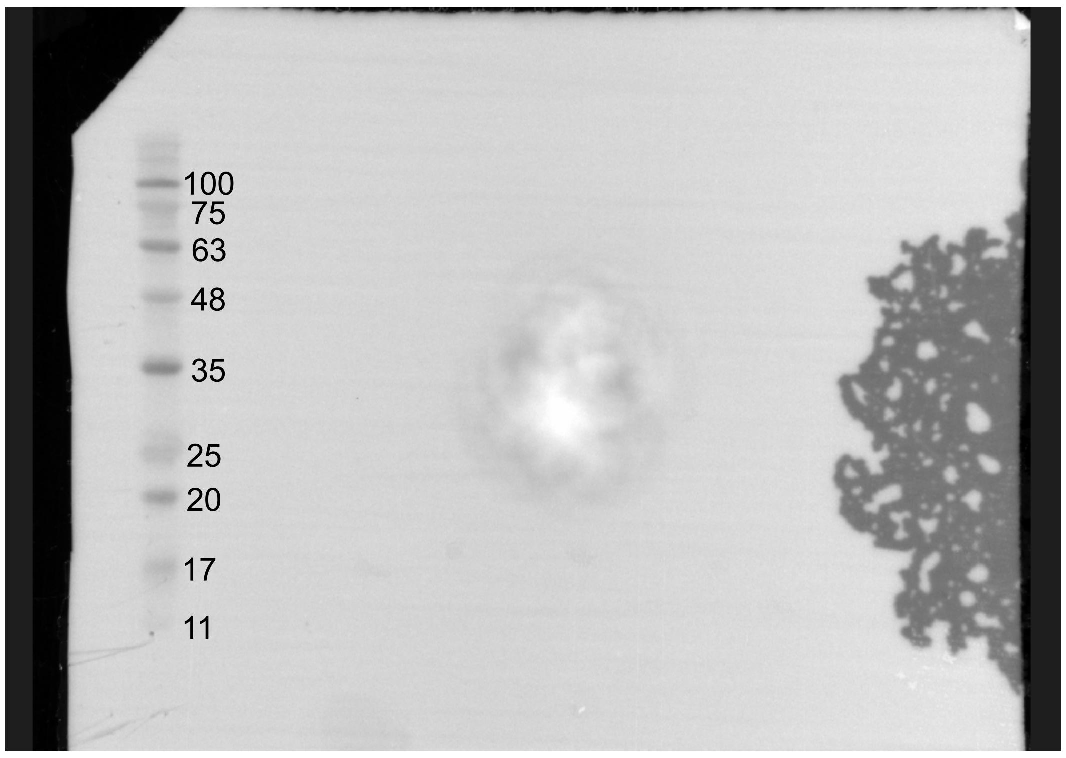

Merged image

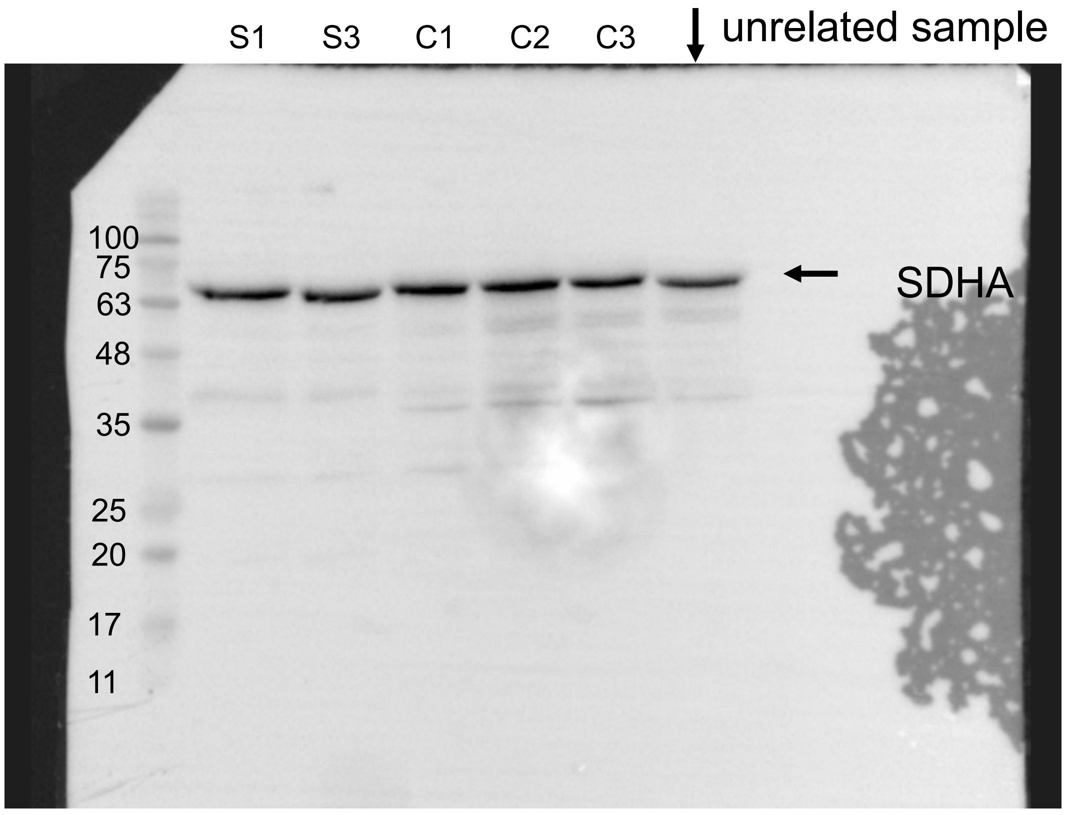

Figure 3A continued

NDUFB8 panel

Chemiluminescent signal used in figure

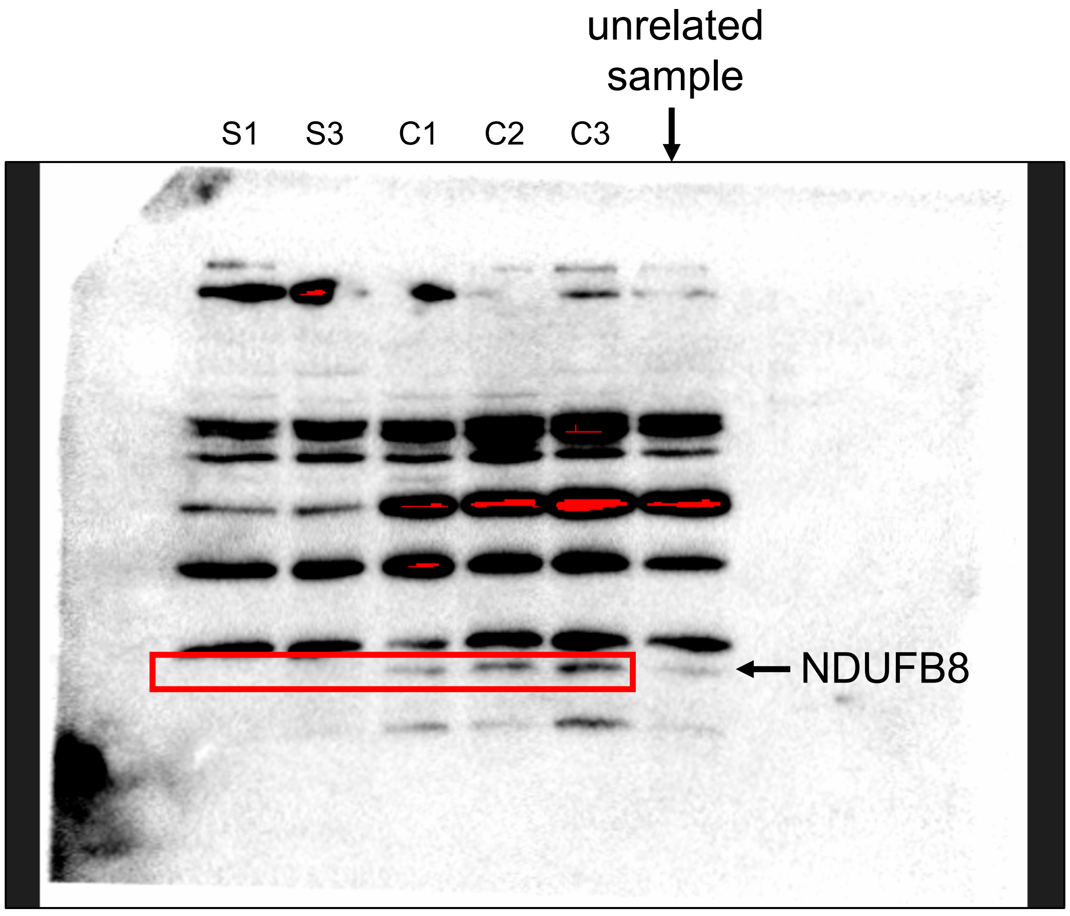

Colourimetric image to show MW marker (kDa)

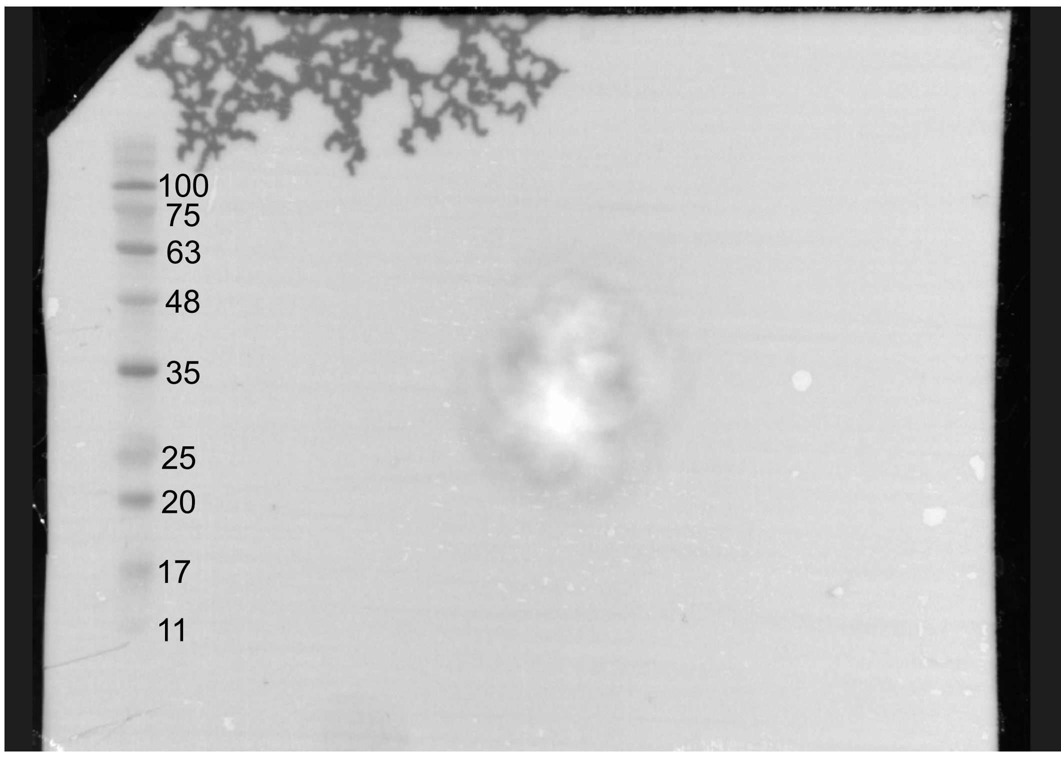

Merged image

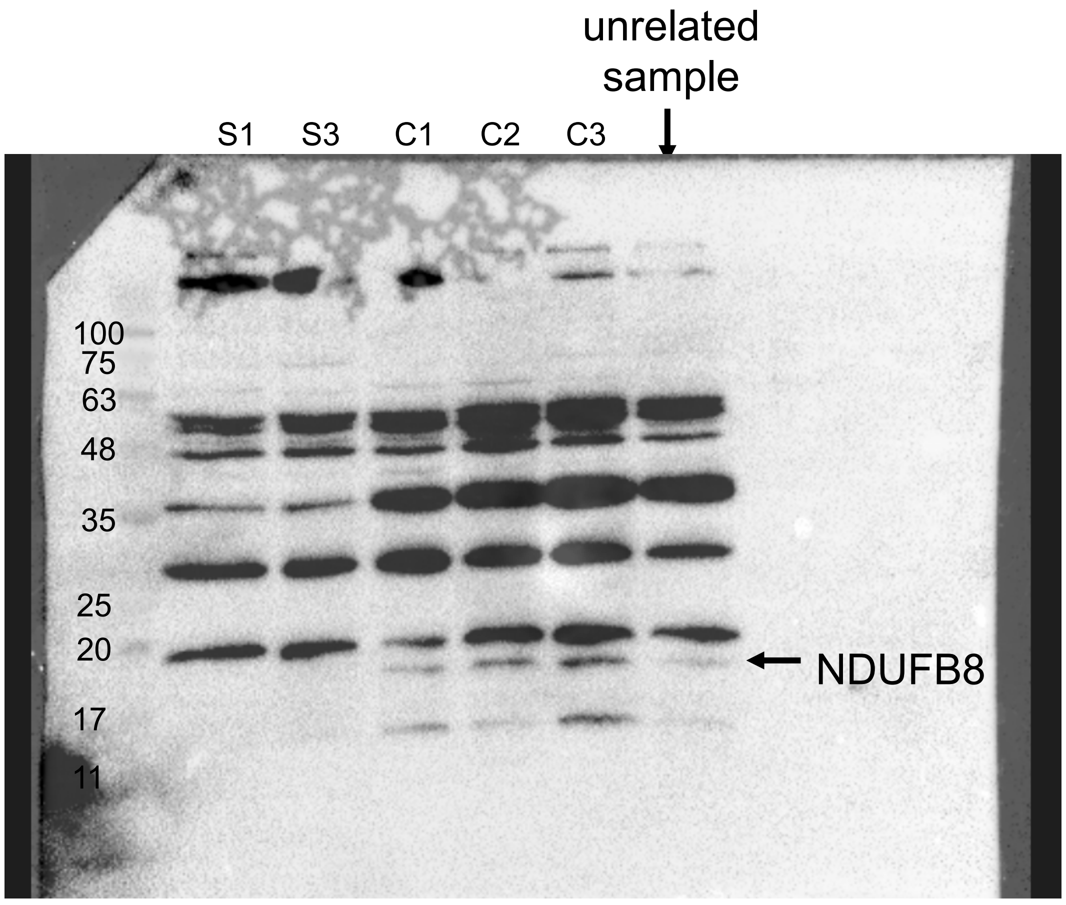

Figure 3B

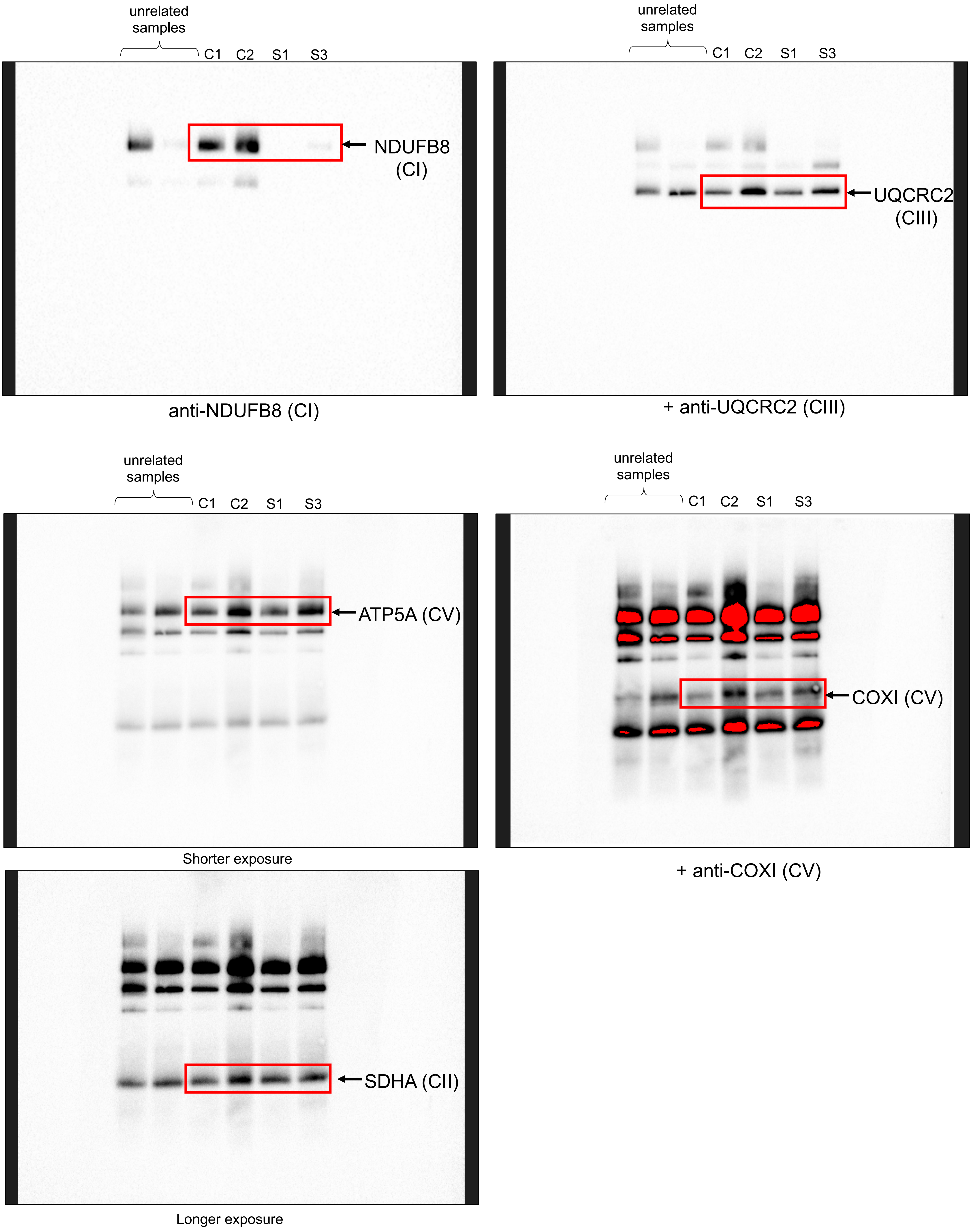

+ anti-SDHA (CII) and anti-ATP5A (CV)

Figure 3C

NDUFC2 panel

Chemiluminescent signal used in figure

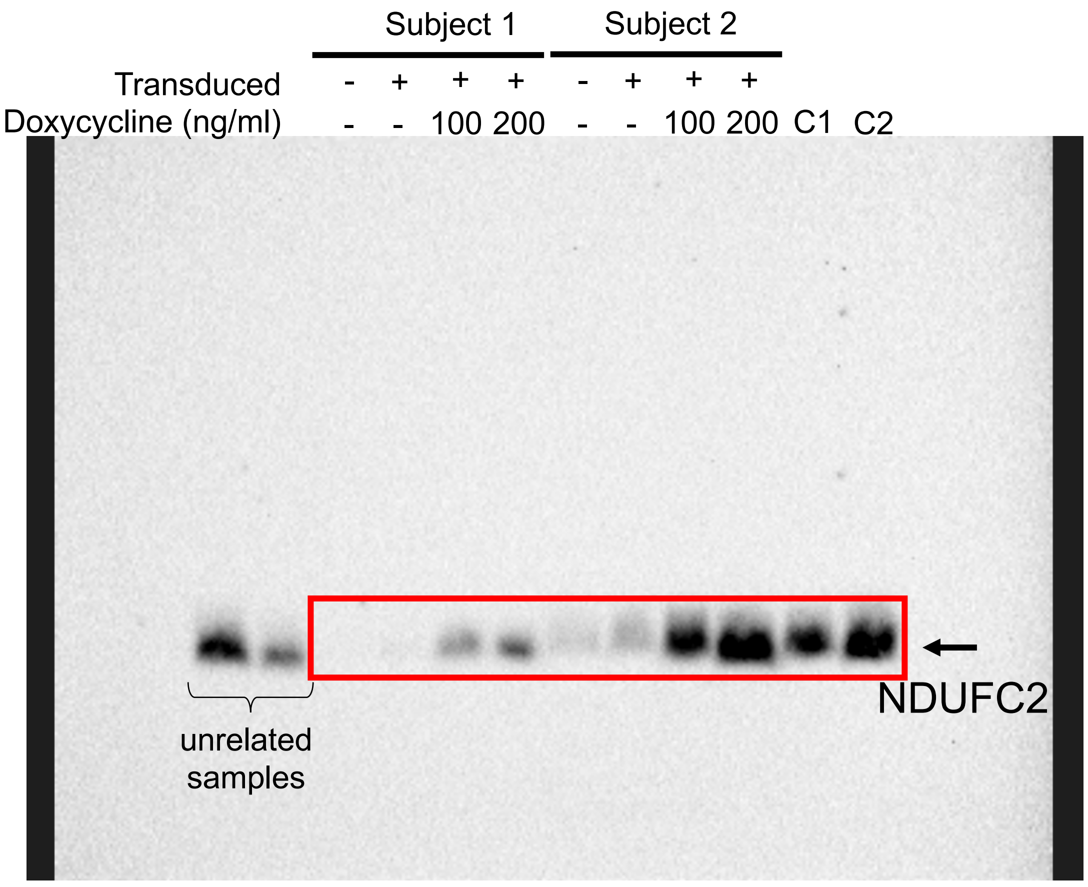

Colourimetric image to show MW marker (kDa)

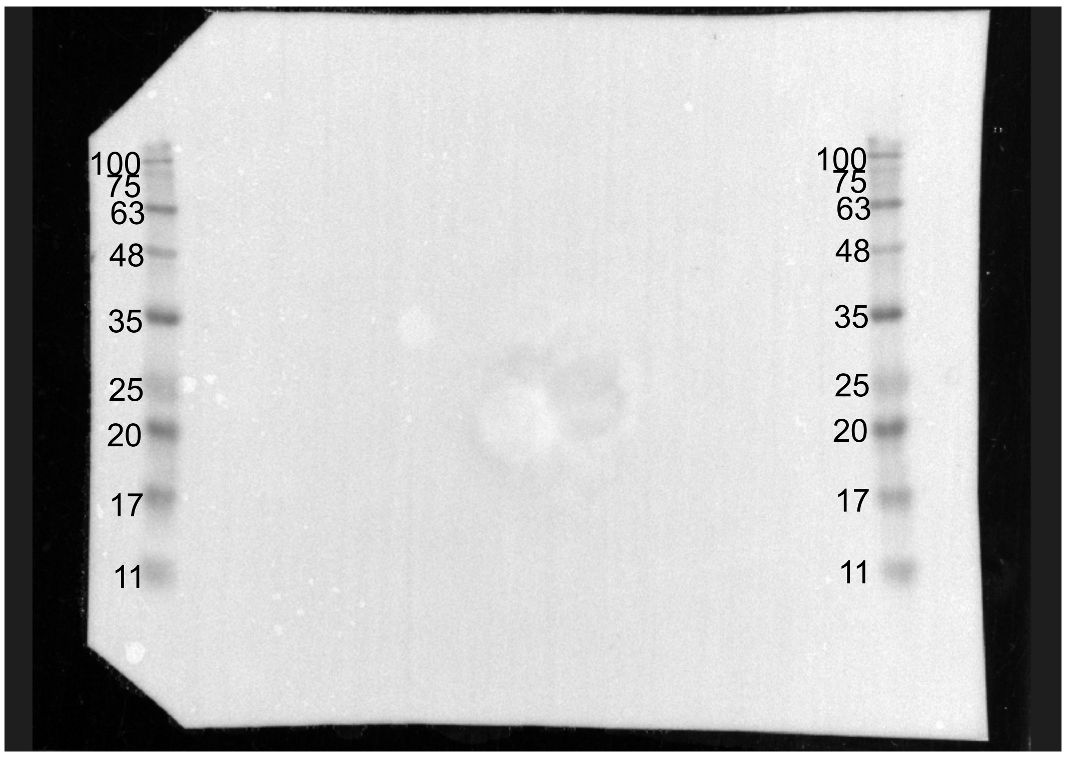

Merged image

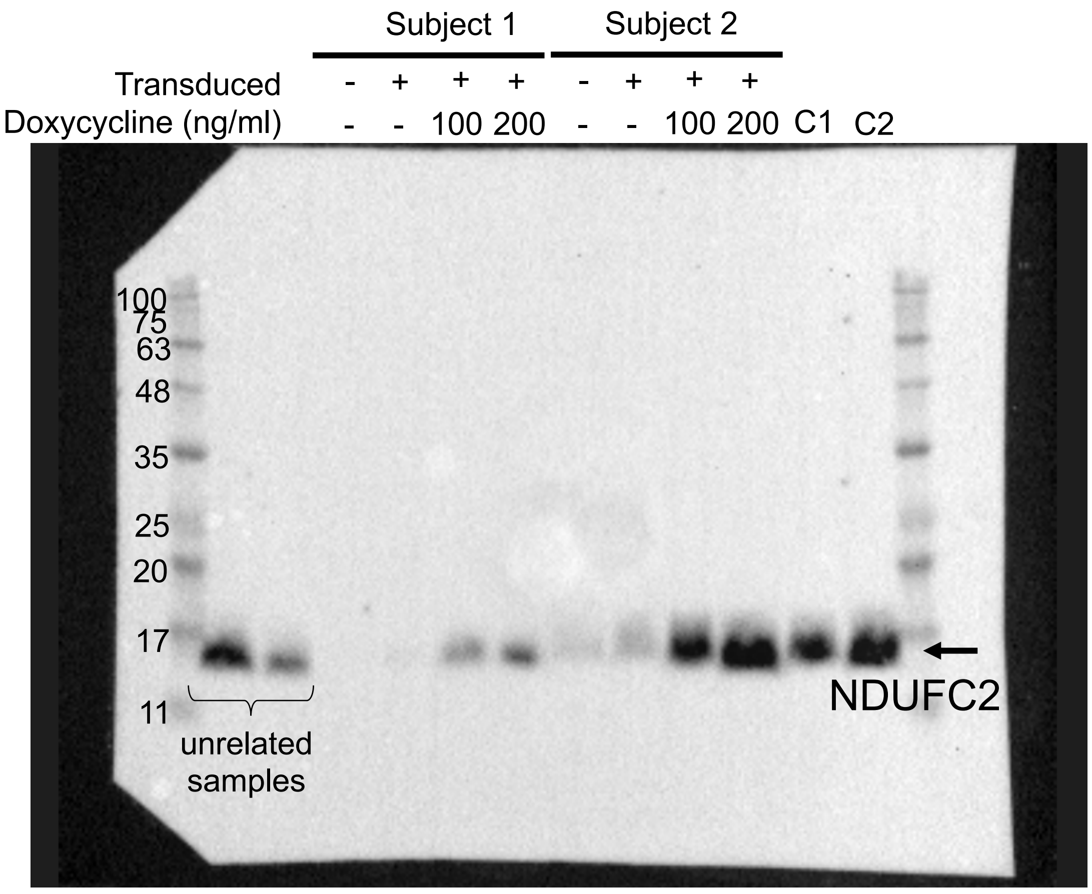

Figure 3C continued

NDUFB8 and NDUFA9 panels

Chemiluminescent signal used in figure

Colourimetric image to show MW marker (kDa)

	Subject 1				Subject 2					
Transduced	-	+	+	+	-	+	+	+		
Doxycycline (ng/ml)	-	-	100	200	-	-	100	200	C1	C2

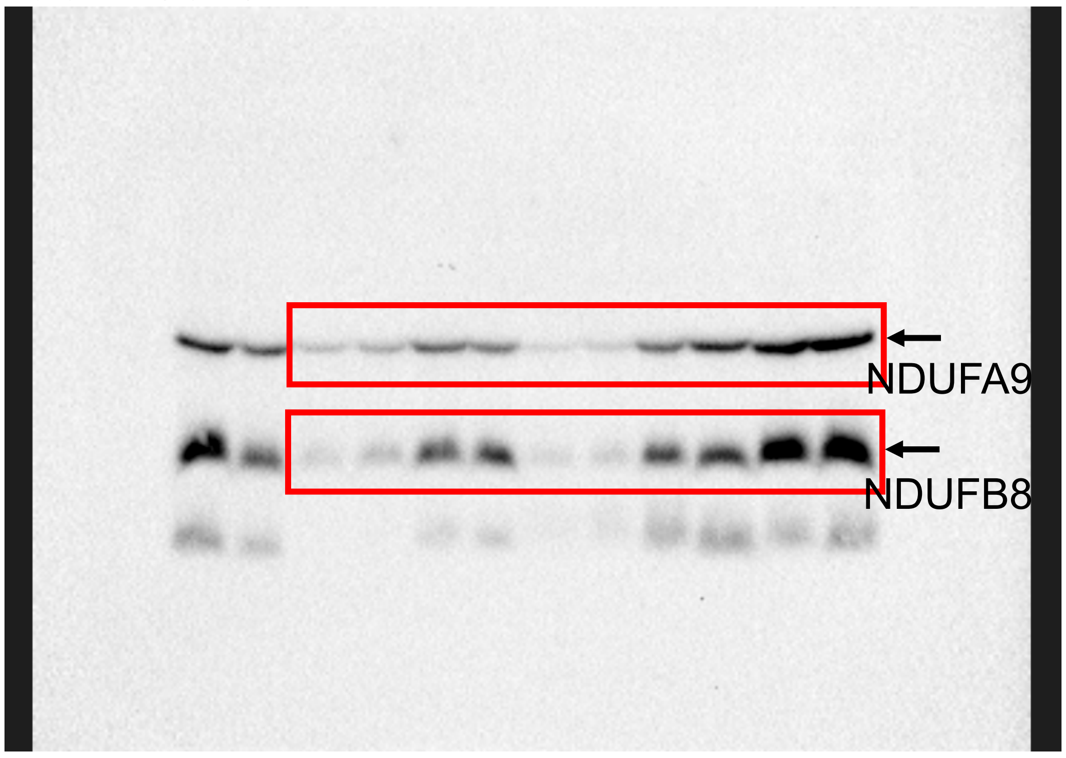

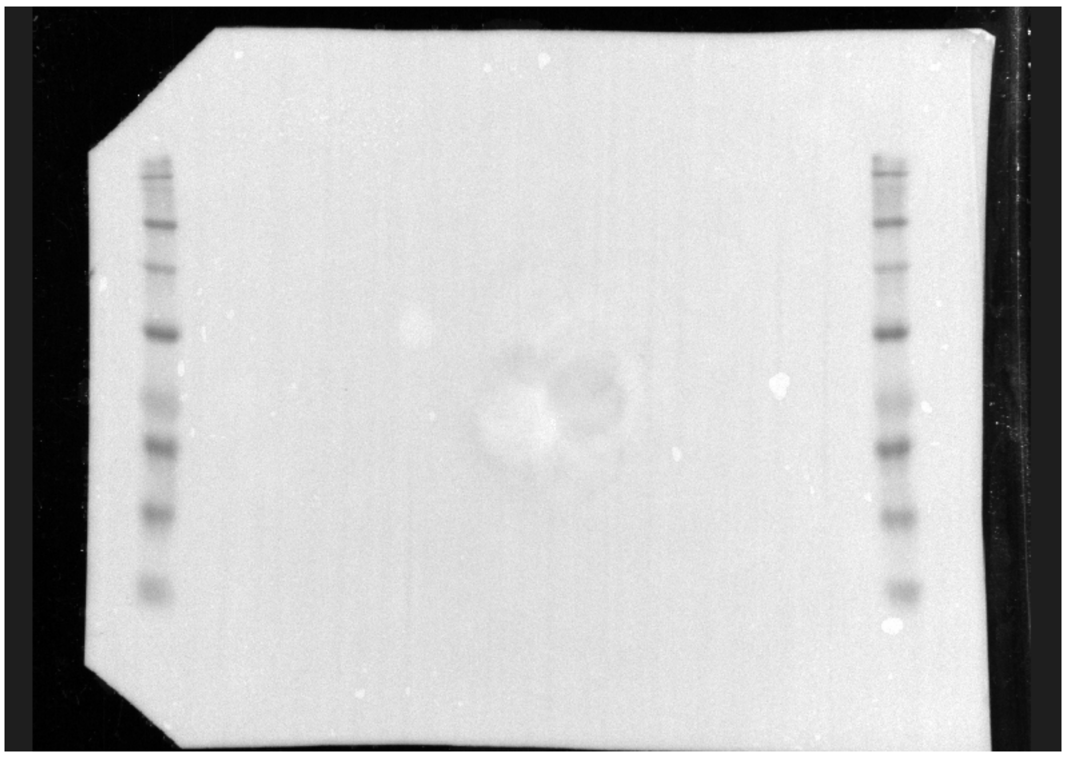

Merged image

	Subject 1				Subject 2					
Transduced	-	+	+	+	-	+	+	+		
Doxycycline (ng/ml)	-	-	100	200	-	-	100	200	C1	C2

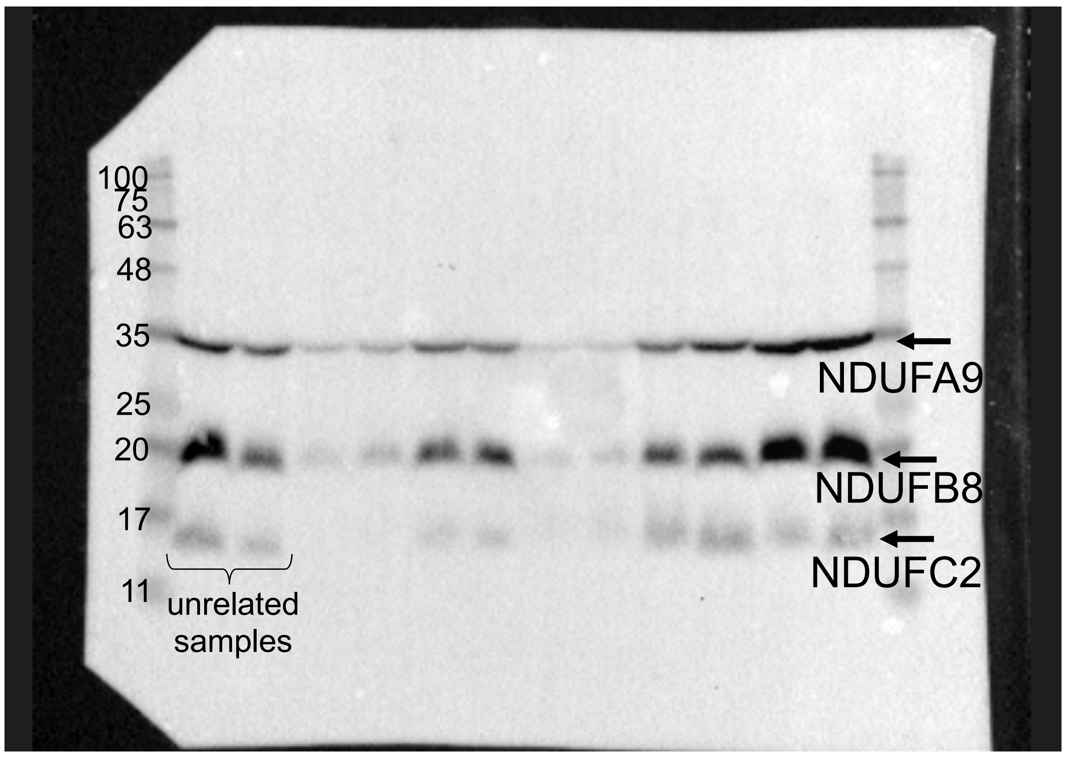

Figure 3C continued

NDUFV1 panel

Chemiluminescent signal used in figure

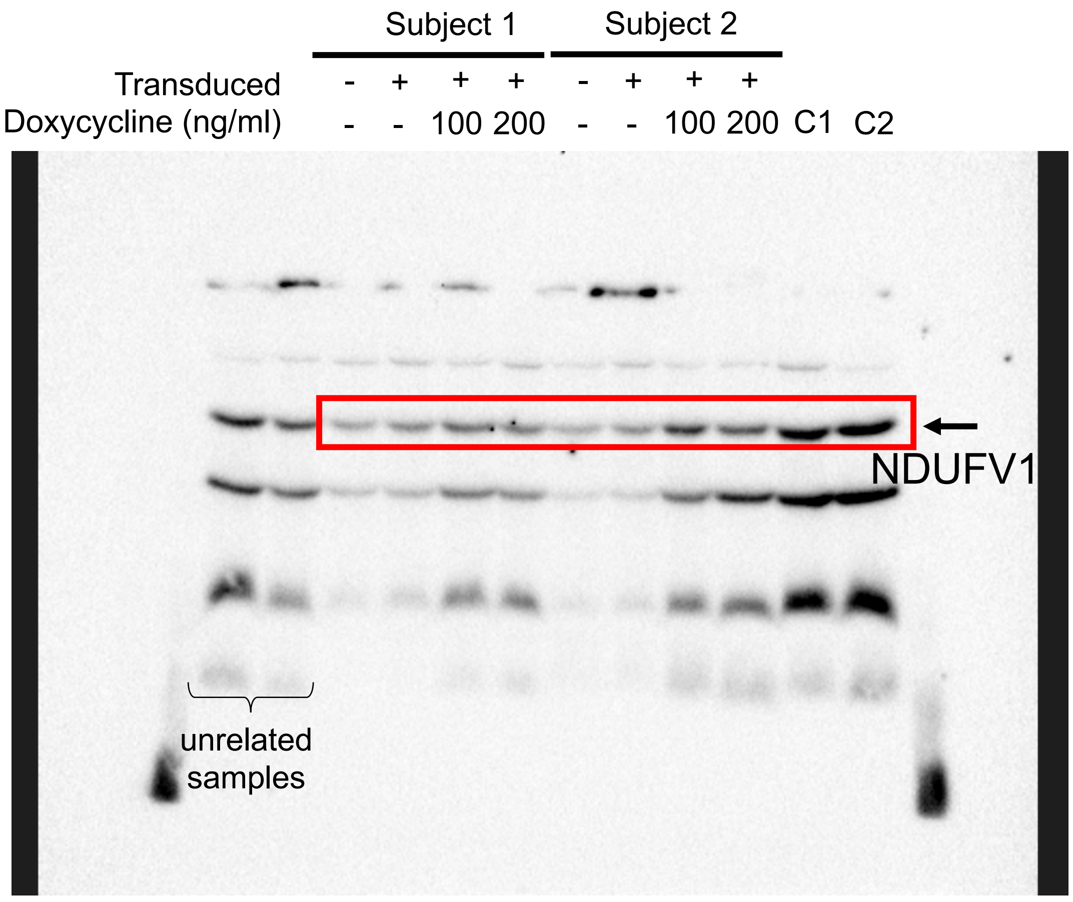

Colourimetric image to show MW marker (kDa)

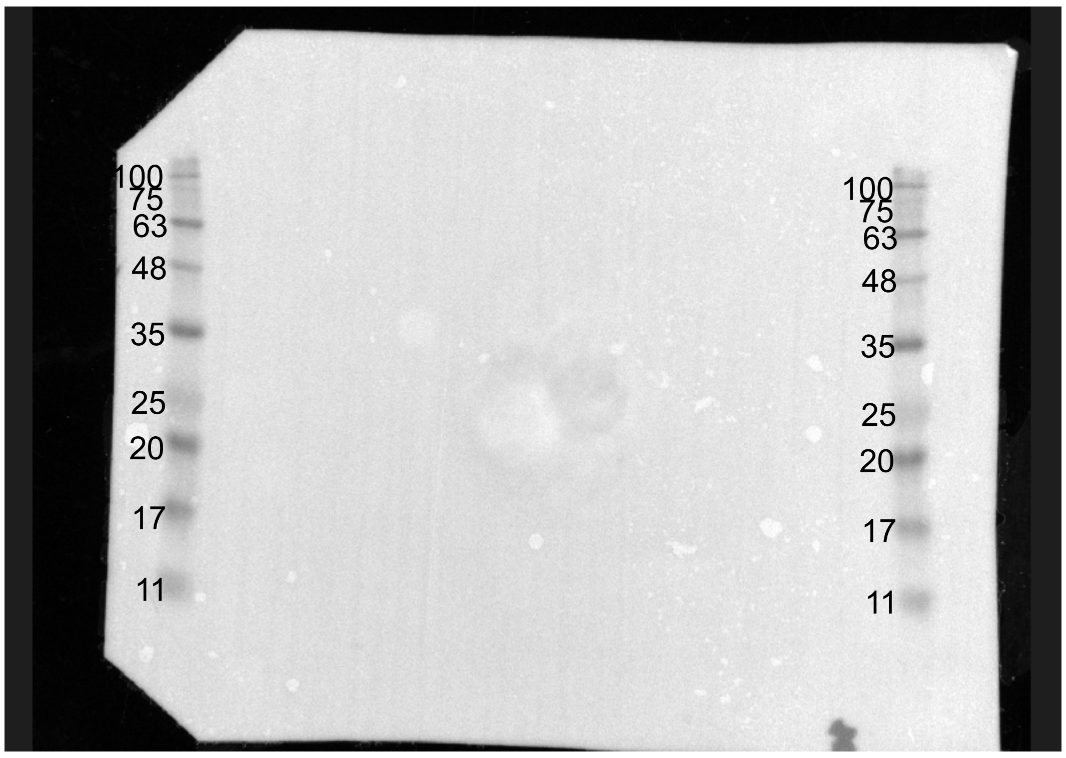

Merged image

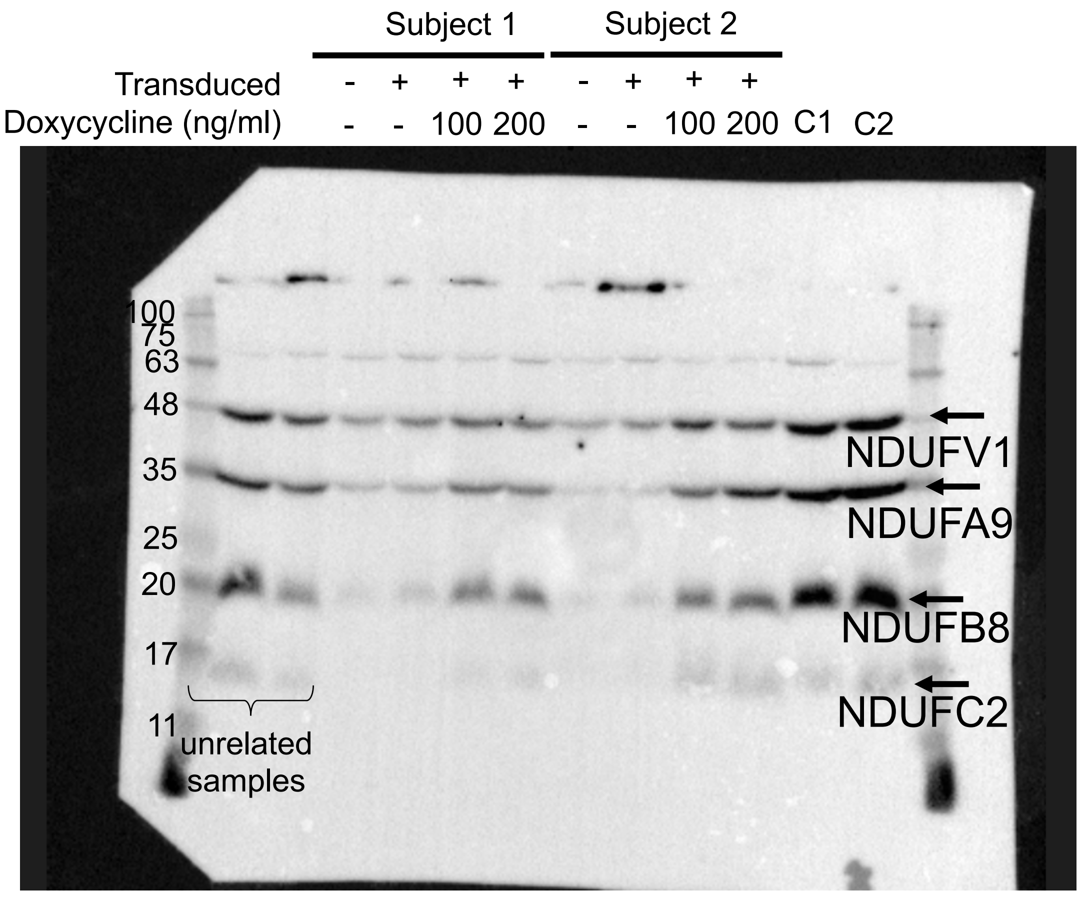

Figure 3C

SDHA panel

Chemiluminescent signal used in figure

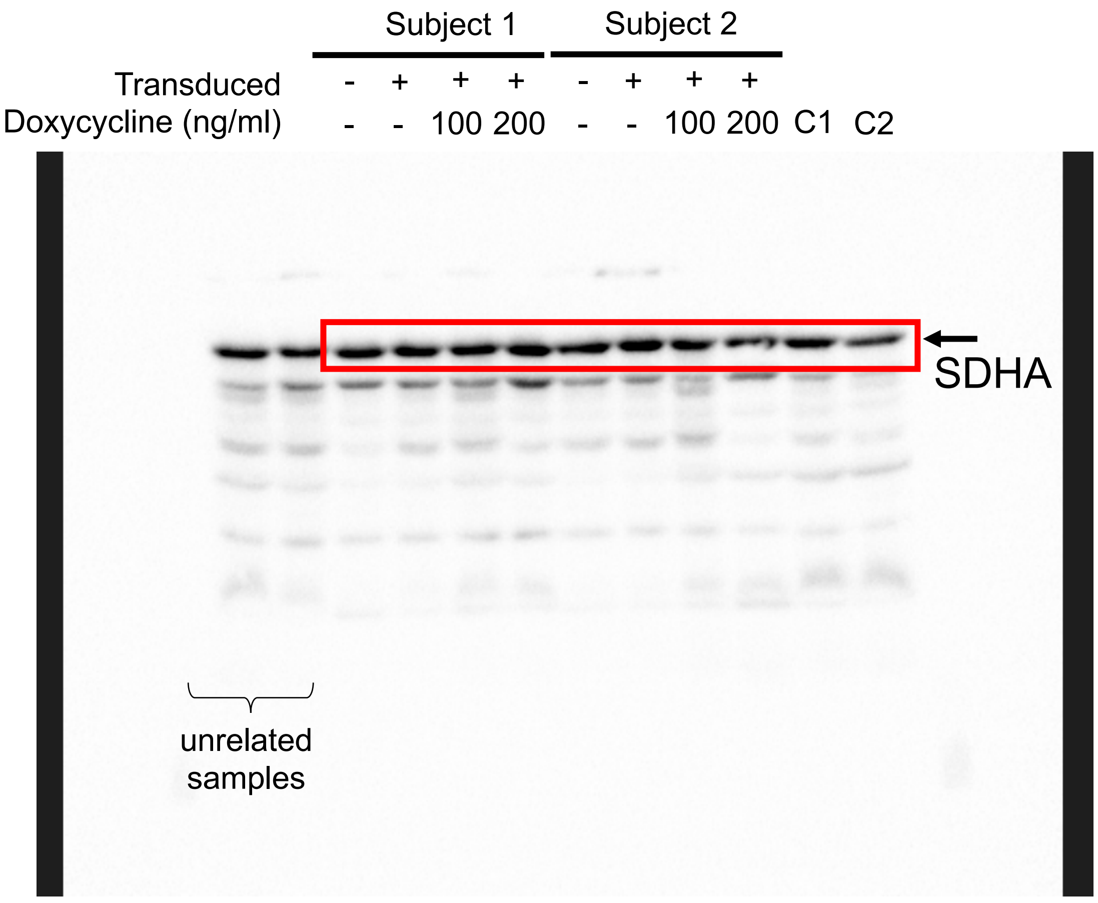

Colourimetric image to show MW marker (kDa)

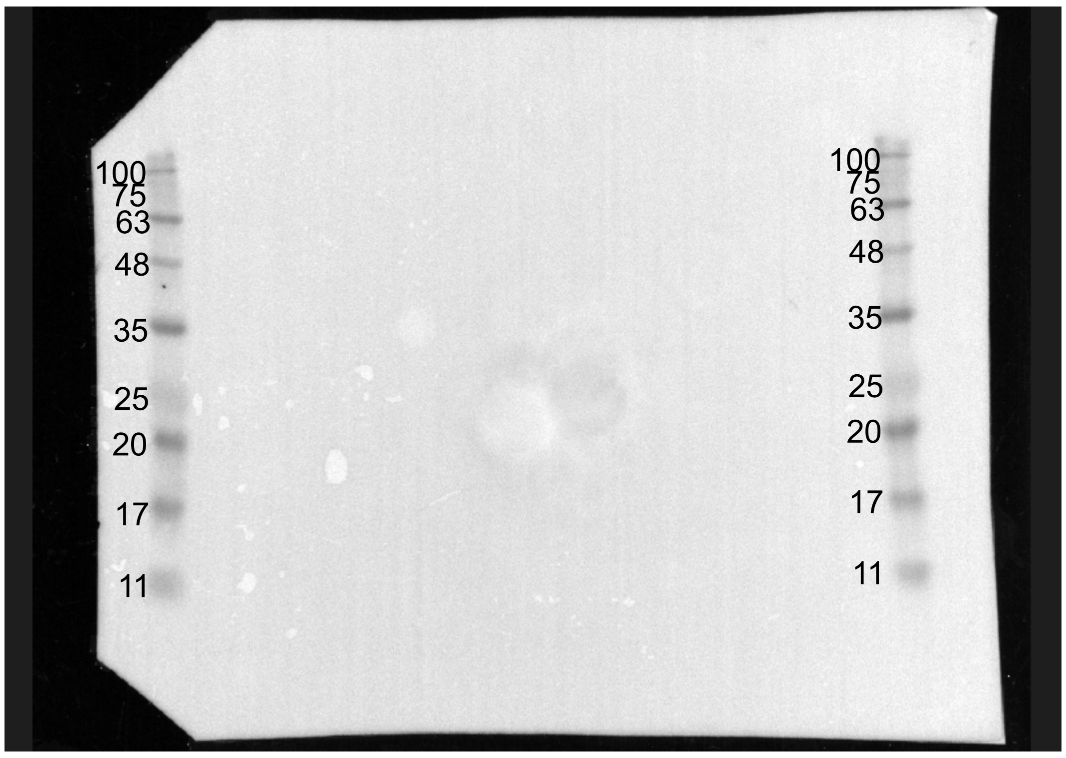

Merged image

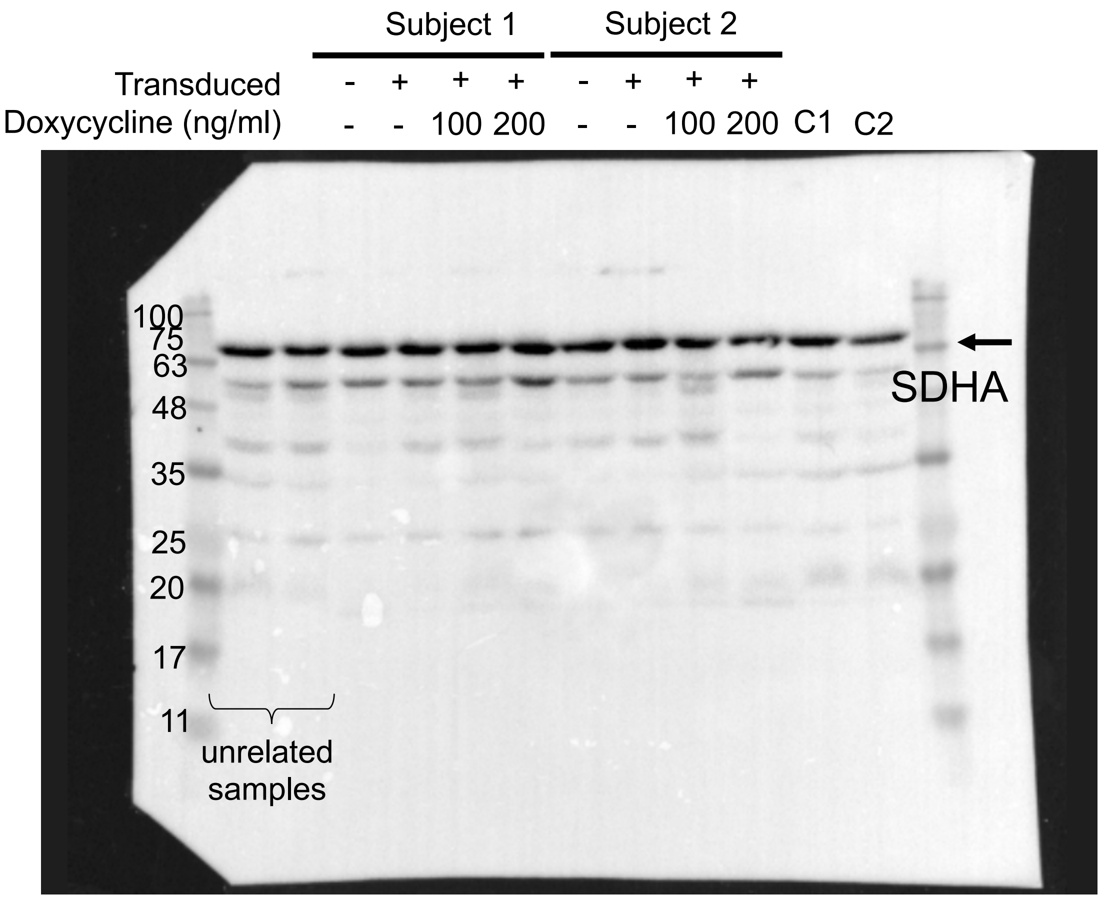

Figure 3D

Subject 1 panel

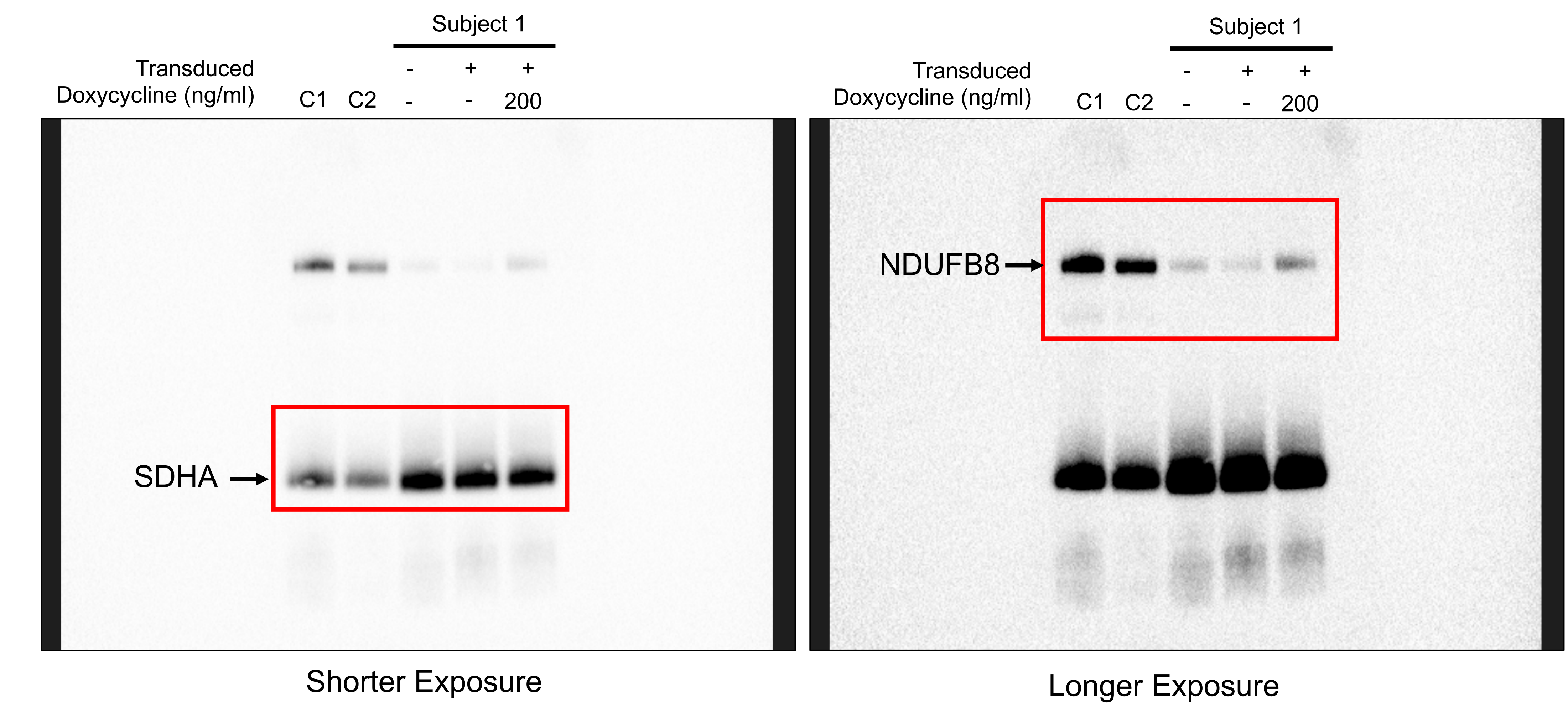

Subject 2 panel

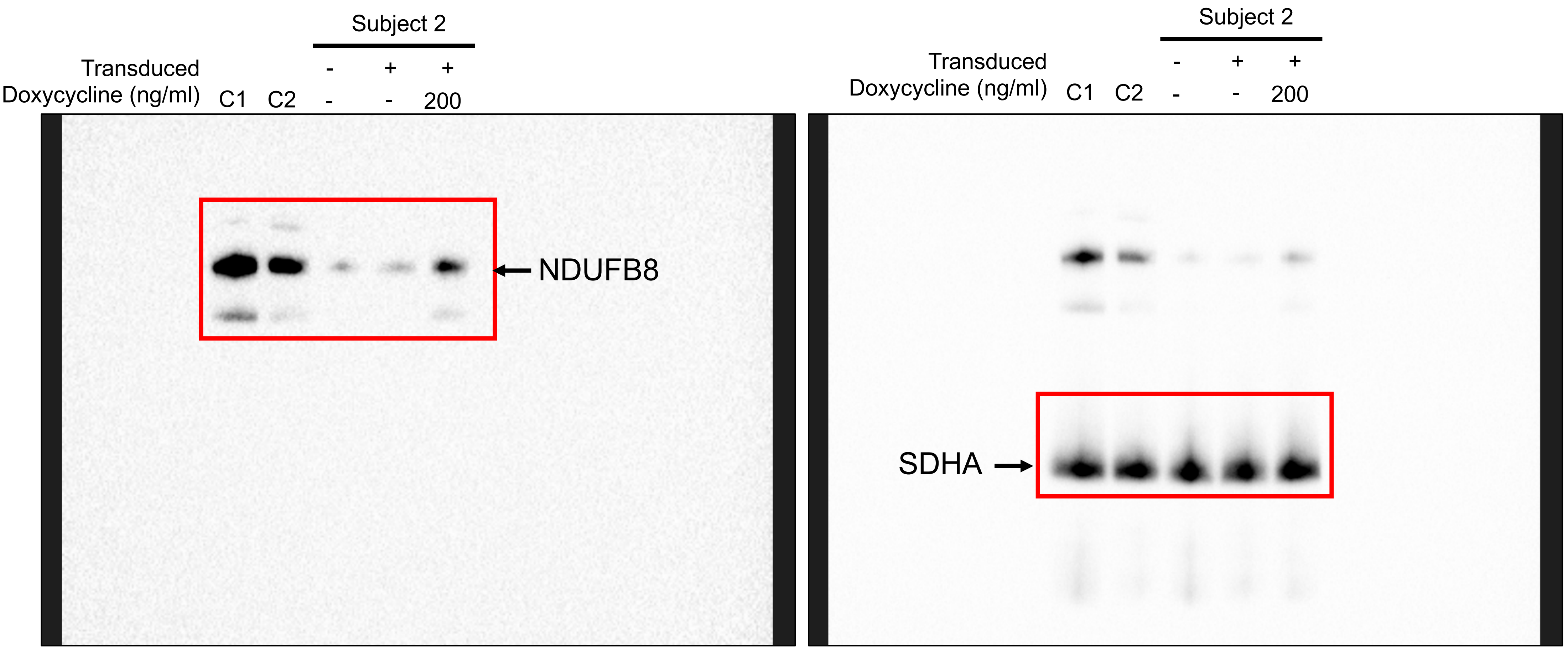

Supplement: Supplementary file 2 — Source Data for Figure 3 [file EMMM-12-e12619-s002.pdf]
